# Deubiquitination of CIB1 by USP14 promotes lenvatinib resistance via the PAK1-ERK1/2 axis in hepatocellular carcinoma

**DOI:** 10.7150/ijbs.96031

**Published:** 2024-06-03

**Authors:** Ming-Hao Xu, Yi-Min Zheng, Bu-Gang Liang, Wen-Xin Xu, Jun Cao, Pei Wang, Zi-Ying Dong, Chen-Hao Zhou, Hui-Chuan Sun, Ning Ren, Ai-Wu Ke, Ying-Hao Shen

**Affiliations:** 1 Department of Liver Surgery and Transplantation, Liver Cancer Institute and Zhongshan Hospital, Fudan University, Shanghai, 200032, China.; 2 Institute of Fudan-Minhang Academic Health System, Minhang Hospital, Fudan University, Shanghai, 200032, China.; 3 Department of Hepatobiliary Surgery, Clinical Medical College, Yangzhou University, Yangzhou, 225009, China.; 4 Department of Digestive Medicine, Wuwei People's Hospital, Gansu, 733000, China.; 5 Department of CT/MRI Center, Wuwei People's Hospital, Gansu, 733000, China.

**Keywords:** USP14, HCC, CIB1, Lenvatinib resistance

## Abstract

**Background:** Lenvatinib is the most common multitarget receptor tyrosine kinase inhibitor for the treatment of advanced hepatocellular carcinoma (HCC). Acquired resistance to lenvatinib is one of the major factors leading to the failure of HCC treatment, but the underlying mechanism has not been fully characterized.

**Methods:** We established lenvatinib-resistant cell lines, cell-derived xenografts (CDXs) and patient-derived xenografts (PDXs) and obtained lenvatinib-resistant HCC tumor tissues for further study.

**Results:** We found that ubiquitin-specific protease 14 (USP14) was significantly increased in lenvatinib-resistant HCC cells and tumors. Silencing USP14 significantly attenuated lenvatinib resistance *in vitro* and *in vivo*. Mechanistically, USP14 directly interacts with and stabilizes calcium- and integrin-binding protein 1 (CIB1) by reversing K48-linked proteolytic ubiquitination at K24, thus facilitating the P21-activated kinase 1 (PAK1)-ERK1/2 signaling axis. Moreover, *in vivo* adeno-associated virus 9 mediated transduction of CIB1 promoted lenvatinib resistance in PDXs, whereas CIB1 knockdown resensitized the response of PDXs to lenvatinib.

**Conclusions:** These findings provide new insights into the role of CIB1/PAK1-ERK1/2 signaling in lenvatinib resistance in HCC. Targeting CIB1 and its pathways may be a novel pharmaceutical intervention for the treatment of lenvatinib-resistant HCC.

## Introduction

Liver cancer is the fourth leading cause of cancer-related death worldwide, and hepatocellular carcinoma (HCC) accounts for more than 80% of liver cancer cases.^1^ The risk factors for HCC include hepatitis B virus, hepatitis C virus, nonalcoholic fatty liver disease and alcohol-related cirrhosis.^2^ The onset of HCC has not been well defined, and when diagnosed, most patients have advanced disease with a poor prognosis and are generally not amenable to curative local treatment.^3^ Sorafenib was the first targeted treatment for advanced HCC, but no other drugs for HCC were approved for use within 10 years of its release.^4^ Lenvatinib is an oral multitarget receptor tyrosine kinase inhibitor (TKI) that inhibits VEGFR1-VEGFR3, FGFR1-FGFR4, PDGFRα, KIT, and RET and was recommended as a first-line treatment for advanced HCC by the Food and Drug Administration (FDA) after a phase III noninferiority clinical trial.^5^ Compared to sorafenib, lenvatinib significantly improved the median time to progression-free survival (mPFS) and objective response rate (ORR) in patients with advanced HCC, but the median overall survival (mOS) time did not improve.^5^ This is largely due to the rapid development of acquired resistance in HCC patients treated with lenvatinib, which limits the long-term survival of HCC patients. Therefore, elucidating the molecular mechanism underlying lenvatinib resistance and reversing lenvatinib resistance are highly clinically important and socially beneficial for improving the survival of patients with HCC.

The ubiquitin proteasome system (UPS) regulates protein degradation in cells through a series of steps, such as substrate recognition, ubiquitin binding, and proteasome degradation. Ubiquitin specific protease 14 (USP14) is the only USP family that can reversibly bind to proteasome 19S regulatory particles and inhibit their degradation by removing the ubiquitin chain from the substrate.^6^ Studies have shown that the USP family is involved in different physiological processes in cells and in the occurrence of many cancers, including oral cancer,^7^ head and neck squamous cell carcinoma,^8^ and gastric cancer^9^. Previous studies have shown that USP14 regulates DNA damage repair in prostate cancer cells by targeting ubiquitin to modify RNF168.^10^ USP14 can also promote tryptophan metabolism and immune suppression via posttranslational regulation of IDO1 in colorectal cancer.^11^ In addition, USP14 reportedly regulates the expression of downstream target genes of TAZ through a feedback mechanism and ultimately promotes pancreatic ductal adenocarcinoma tumor progression and liver metastasis.^12^ In recent years, it has been found that USP14 acts as an oncogene, promotes the formation of HCC, and can be used as a molecular marker of HCC cells.^13^, ^14^ However, the effect of USP14, a deubiquitinating enzyme, on the treatment resistance of HCC has yet to be explored.

Calcium- and integrin-binding protein 1 (CIB1) can specifically bind to the cytoplasmic domain of integrin αIIb in platelets.^15^ Initial reports suggest that CIB1 is fixed to the platelet membrane by myristoylation. After platelet activation, CIB1 is concentrated in the filamentous foot through its association with the cytoskeleton.^16^, ^17^ As our understanding of intracellular interactions has progressed, CIB1 has been reported to interact with a variety of proteins with different functions, such as kinases and phosphatases. CIB1 is involved in various processes, such as spermatogenesis, thrombosis, cardiac hypertrophy, and angiogenesis.^18^, ^19^ Growing evidence also points to a new role for CIB1 in various cancers. The study of CIB1 indicated that CIB1 can play a role in cell migration, proliferation and survival in cancer cell lines and in tumor growth *in vivo* in breast cancer.^20^ Researchers have also shown that CIB1 is essential for promoting HCC cell proliferation and that CIB1 levels in clinical liver cancer tissues are significantly higher than those in marginal tissues from the center of the tumor mass.^21^ In the tumor microenvironment, tumor growth and survival depend not only on carcinogenic signaling pathways but also on the formation of new blood vessels. It has also been reported that CIB1 can induce angiogenesis in tumors, thereby promoting tumor progression and treatment resistance.^22^ However, whether CIB1 functions during treatment resistance in HCC has not been determined.

In the present study, we found that USP14 expression was significantly increased in lenvatinib-resistant cells, resistant cell-derived xenografts (CDXs) generated by continuous administration *in vivo* and in clinical patients with lenvatinib resistance. Silencing USP14 significantly reversed lenvatinib resistance *in vitro* and *in vivo*. In addition, USP14 stabilized CIB1 through its deubiquitinating function, which in turn facilitated the PAK1-ERK1/2 signaling axis and induced lenvatinib resistance in HCC cells. Based on these results, we propose a novel mechanism for lenvatinib resistance in HCC, providing new clues for improving the efficacy of targeted therapy in patients with advanced HCC.

## Materials and methods

### Patients and tissue samples

We studied two cohorts of patients with HCC who underwent surgery at Zhongshan Hospital, Fudan University. These cohorts included randomly selected cohort 1 (with 170 samples) and cohort 2 (with 50 samples). HCCs in cohort 2 received monotherapy or combination therapy with lenvatinib. HCC was diagnosed using standard imaging techniques either in the presence or absence of elevated serum tumor markers. Patients were considered inoperable before the initiation of systemic treatment if they had advanced-stage HCC, insufficient remnant liver volume (< 40% of the standard liver volume for patients with liver cirrhosis or < 30% of the standard liver volume for patients without liver cirrhosis) or outside up-to-seven criteria.^23^ Tumor response was evaluated by abdominal contrast-enhanced MRI or CT according to the Response Evaluation Criteria in Solid Tumors (RECIST) v1.1. Those evaluated as partial response (PR) or complete response (CR) were considered lenvatinib sensitive, and those evaluated as stable disease (SD) or progressive disease (PD) were considered clinically resistant to lenvatinib. Other treatment information and dosing regimens were described in detail in our previous research.^24^ The gene expression data for GSEA were downloaded from http://www.cbioportal.org for the TCGA cohort.

All human samples were anonymously coded in accordance with local ethical guidelines (as stipulated by the Declaration of Helsinki). Written informed consent was obtained from each patient, and the study protocol was approved by the Review Board of Zhongshan Hospital, Fudan University.

### Cell lines and cell culture

The human HCC cell lines PLC/PRF/5 and Huh7 were chosen based on a previous study, which demonstrated that PLC/PRF/5 and Huh7 exhibited different degrees of lenvatinib resistance.^25^ The cell culture media used were prepared as recommended by the supplier. After supplementation with 10% fetal bovine serum (FBS), the cells were incubated in chambers maintained in an atmosphere of 5% CO2 and 95% air at 37°C. PLC-R and Huh7-R cells with stable USP14 knockdown and PLC-C cells with stable USP14 overexpression were generated by lentiviral infection. The CIB1 plasmid and PAK1 plasmid was purchased from Shanghai Genechem Co., Ltd. For siRNA transfection, cells were plated at 30-60% confluence in Opti-MEM serum-free medium and transfected with a specific siRNA duplex using Lipofectamine RNAiMAX Reagent Agent (Life Technologies) according to the manufacturer's instructions. For plasmid transfections, cells were grown to 60% confluence in 6-cm dishes and transfected with 4 μg of plasmid using 4μL of Lipofectamine 3000 (Invitrogen) according to the manufacturer's instructions. For experiments using USP14 truncations (Δ201220), 10 μm of MG-132 was added 6 h prior to the harvest of the cells. The oligo sequences of the shRNAs used are listed in Table S5.

### Lenvatinib treatment on patients, cells and mice models

Lenvatinib was used according to the manufacturer's instructions. Briefly, patients with a body weight less than 60 kg received 8 mg/d lenvatinib, while patients with a body weight greater than 60 kg received 12 mg/d lenvatinib. For CDXs and PDXs, lenvatinib was administered at a dosage of 20 mg/kg/d by gavage based on the literature^26^ and our preliminary experiments. For cell cultures, the concentration was defined as the IC50 value of the corresponding cell line with indicated treatment.

### Immunohistochemistry (IHC) and immunofluorescence (IF)

Antihuman rabbit monoclonal antibodies against USP14 (ab192618, ABCAM) and antihuman rabbit polyclonal antibodies against CIB1 (ab220606, ABCAM) were used as primary antibodies to detect the expression of USP14 and CIB1. Briefly, paraffin-embedded sections were dewaxed in xylene and then hydrated with ethanol, after which the concentration was decreased. After that, the slides were incubated in a buffer bath, heated for 15 minutes to extract the antigen, and then blocked with 3% hydrogen peroxide in methanol at 37°C for 30 minutes. The slides were incubated with the primary wash agent and applied antibodies, placed in a humidified 4℃ chamber overnight, and stained with DAB reagent (Dako REALTM EnVisionTM Detection System, Denmark). The slides were counterstained with hematoxylin, dehydrated, and mounted. Finally, the tumor slides were scanned with a 20x view of 1.44 mm2 by a MAGSCAN KF-PRO-120. The expression of USP14 and CIB1 in three individual fields of the slides was evaluated by the H-score method, after which the mean values were calculated. The other antibodies used can be found in Table S6.

For IF, cells cultured on coverslips were fixed with 4% formaldehyde (10 min), permeabilized with 0.3% Triton X-100 (10 min, Beyotime, Shanghai, China), and blocked with 5% BSA (60 min) at 37°C. The cells were treated with the primary antibody overnight at 4°C and then with secondary antibodies conjugated with Alexa Fluor-488 or -594 (60 min, Abcam) as recommended. Then, the coverslips were washed with PBS, stained with 4′,6-diamidino-2-phenylindole (DAPI; Yeasen, Shanghai, China), and evaluated using laser confocal microscopy (Leica TCS SP5 II, Wetzlar, Germany). For mIF, Primary antibodies were incubated for overnight at 4°C, followed by incubation with the corresponding secondary horseradish peroxidase-conjugated antibody. The slides were again placed in citrate buffer to remove redundant antibodies before the next step. Finally, the slides were incubated with DAPI solution at 37℃ for 10 min in the dark, and evaluated using laser confocal microscopy (Leica TCS SP5 II, Wetzlar, Germany).

### Quantitative real-time PCR and western blot

Total RNA was extracted using TRIzol reagent (Invitrogen). Quantitative real-time PCR (qRT-PCR) was performed using the StepOnePlus Real-time PCR system (Applied Biosystems) with SYBR Green PCR Master Mix (Takara, China) according to the manufacturer's protocol. The sequences of the primers used for PCR are listed in Table S5. These assays were repeated at least three times, and the relative gene expression was determined using the 2-ΔΔCt method.

For Western blotting, protein was extracted from HCC cells using RIPA buffer, and the results were determined using a BCA protein assay kit (Beyotime Biotechnology). The extracted proteins (40 μg per lane) were separated via SDS-polyacrylamide gel electrophoresis, and the separated proteins were subsequently transferred to polyvinylidenedifluoride (PVDF) membranes (Bio-Rad). The PVDF membranes were blocked using 5% nonfat milk for 1 h and primary antibodies. The membranes were labeled with peroxidase secondary antibodies. An ECL detection system (Thermo) was used for visualization. The antibodies used for Western blotting, immunohistochemistry and immunofluorescence are listed in Table S6.

### CCK-8 assay

A CCK-8 assay was performed to determine cell viability. Briefly, 1,000 cells/100 μl of DMEM were seeded in each well of a 96-well flat-bottomed plate. At each time point, 10 μL of CCK-8 solution (Yeasen) was added to the sextuplicate wells. The plate was subsequently incubated for 3 h at 37°C, after which the optical density was measured at 450 nm using a multimode microplate reader (Thermo Fisher Scientific, Waltham, MA, USA).

### Mass spectrometry and coimmunoprecipitation (co-IP) assay

To perform the immunoprecipitation (IP) assay, cells were first collected using western/IP lysis buffer supplemented with PMSF and protease and phosphatase inhibitor cocktails. After the sediment was removed by centrifugation at 12,000 × g, the supernatant was precleared using protein A/G-magnetic beads (MedChemExpress, Monmouth Junction, NJ, USA). Subsequently, the precleared lysates were incubated with primary antibodies overnight at 4°C. Protein A/G-magnetic beads were then added to the lysates for 2 h at 4°C. The beads were washed with western/IP lysis buffer, resuspended in SDS-PAGE loading buffer, boiled, and loaded onto 10% gradient gels. Mass spectrometry was subsequently performed (Bioprofile, Shanghai, China). The proteins of interest were detected by Western blotting.

### Flow cytometry

Cells were cultured in 6-well plates. At 72 h after transfection, the cells were collected and washed with cold PBS. The cells were resuspended in staining buffer and examined with an Annexin V-FITC/PI Apoptosis Detection Kit (Yeasen, Shanghai, China) in the dark for 15 min. The samples were detected with a Fortessa (BD) and analyzed with FlowJo V10.6.

### Xenograft tumor model

All mice were housed in a specific pathogen-free facility located in the Laboratory Animal Center of Zhongshan Hospital, Fudan University. The animal experiment protocol received approval from the Animal Care and Use Committee of Fudan University (DSF-2020-064). PLC/Huh7 cells (4×10^6^ cells resuspended in 100 µL of PBS) were injected subcutaneously to induce the growth of subcutaneous tumors in nude mice. Subcutaneous tumor volumes were measured using calipers to calculate the length and width, and the formula (length × width^2^)/2 was used for volume calculation. Mice were euthanized if they displayed signs of distress or when the maximum tumor diameter exceeded 2 cm. For the orthotopic tumor model, a suspension of 20 µL of PLC/Huh7 cells mixed with Matrigel was prepared and injected into the liver using a microinjector. Adequate analgesia was administered during and after surgery. For the PDX model, tumors from HCC patients were minced into pieces (3 × 3 × 3mm) and transplanted into subcutaneous tissue under aseptic conditions. Once the subcutaneous tumor reached a diameter of 1 cm, it was further minced into smaller pieces and then subcutaneously implanted into the flanks of 4- to 5-week-old NOD/SCID mice.

### Statistical analysis

All the statistical analyses were conducted with SPSS software (version 23.0; IBM), R software (version 3.6.3) and GraphPad Prism (version 8.0). OS and DFS were plotted by the Kaplan‒Meier method and evaluated by the log-rank test. Multivariate Cox proportional hazards regression was performed in a stepwise manner. Continuous variables were compared by Student's t test, and the Pearson chi-square test was used to analyze categorical variables. Pearson's correlation analysis was performed to evaluate the correlation between two variables. Three independent experiments with three technical repetitions were performed. A two-tailed P < 0.05 was considered to indicate statistical significance. *p* values are denoted as follows: *p < 0.05, **p < 0.01, and ***p < 0.001.

## Results

### USP14 is associated with lenvatinib resistance

**Figure 1:**
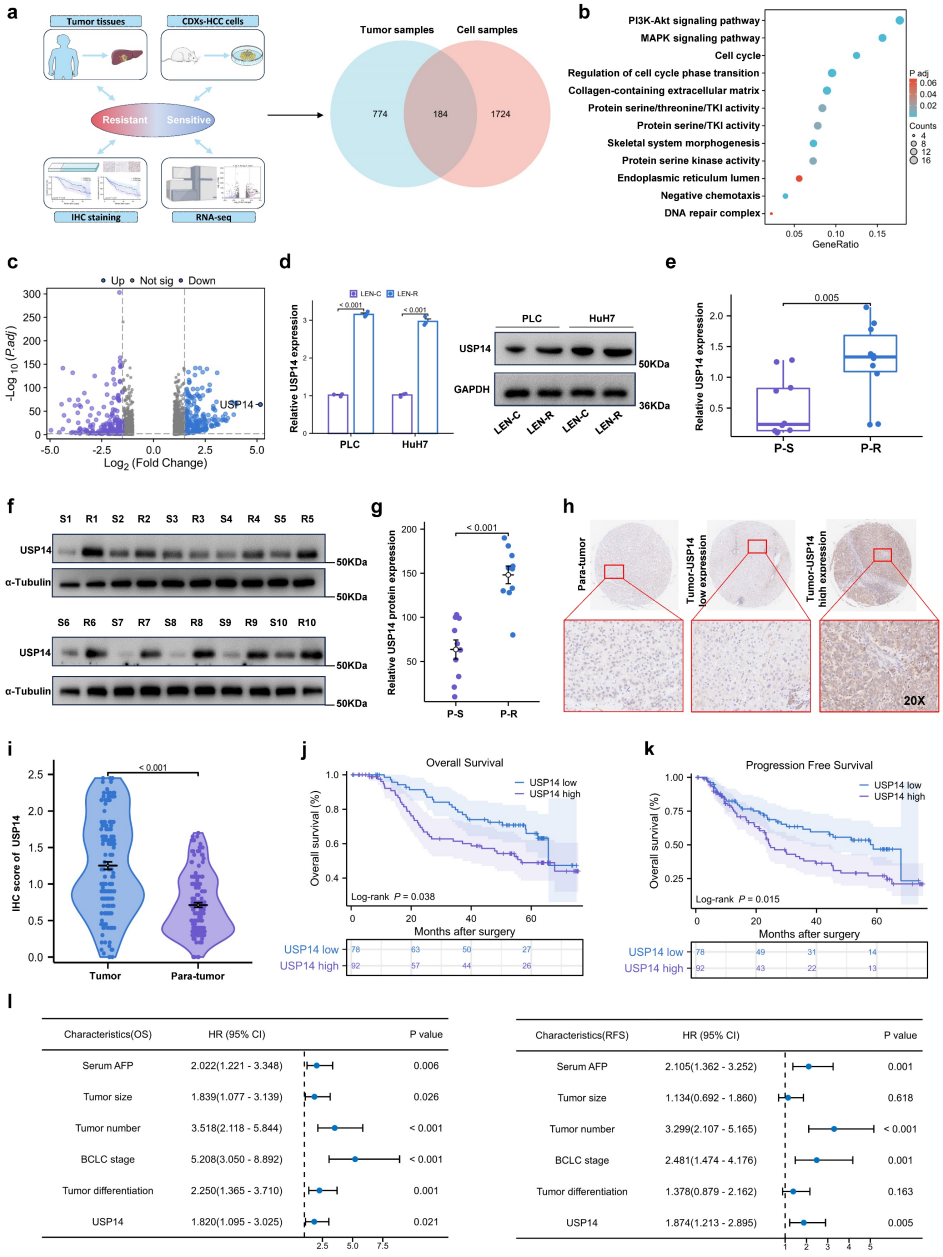
USP14 is associated with lenvatinib resistance. **a.** A Venn diagram was generated from the gene sets enriched for transcripts between lenvatinib-resistant tumor samples and cell samples. Identification of differentially expressed genes by RNA sequence in lenvatinib-resistant cell lines and patients compared to the corresponding sensitive or control Groups **b.** Gene Ontology analysis of differentially expressed genes. **c.** Volcano plots showing the genes differentially expressed between tumor cells and normal cells.** d.** The expression of USP14 in the LEN-R and LEN-C cell lines was determined by qPCR and Western blotting. **e-g.** The expression of USP14 in 10 pairs of lenvatinib-resistant and lenvatinib sensitive patients was determined by qRT-PCR and Western blotting. **h-i.** IHC staining of USP14 expression in 170 HCC patients determined by immunohistochemistry. **j-k.** K‒M survival analysis of HCC patients after resection with low or high USP14 expression. The median USP14 expression was used as the cutoff for low and high USP14 expression. **l.** Forest plot showing the results of multivariate analysis of factors associated with OS and DFS. Three independent experiments with three technical repetitions were performed. Student's *t* test was used for statistical analyses. LEN-R, lenvatinib resistant; LEN-C, lenvatinib control; P-S, lenvatinib-sensitive patient; P-R, lenvatinib-resistant patient. *p* < 0.05 was considered to indicate statistical significance. * *p* < 0.05, ** *p* < 0.01, *** *p* < 0.001.

Two models of lenvatinib-resistant HCC cell lines (Huh7-R and PLC-R) were established through cell xenotransplantation via continuous intragastric administration of lenvatinib (20 mg/kg/d) for three months. During this time, once the tumor diameter reached 1.5cm, the nonnecrotic parts of the subcutaneous tumors were extracted and xenografted into a new batch of nude mice (Fig. S1a). Totally, we performed three cycles of establishing lenvatinib-resistant HCC cell lines *in vivo*. Compared with those in potentially lenvatinib-resistant cells, the cell lines in the control group had significantly lower IC50 value after exposure to lenvatinib, which demonstrated that the Huh7-R and PLC-R cell lines we constructed developed resistance to lenvatinib (Fig. S1b). In addition, compared with control cells, resistant cells had significantly increased colony-forming capacity and reduced apoptosis in response to sustained exposure to lenvatinib (Fig. S1c-d). In addition, LEN-R CDXs had lower sensitivity to lenvatinib treatment than LEN-C CDXs (Fig. S1e). Pretreatment biopsy specimens from 5 patients with lenvatinib-resistant HCC were obtained. Pre- and posttreatment images and the timing of sample collection and lenvatinib administration are shown in Fig. S1f-g. RNA sequencing was subsequently performed to identify critical targets involved in lenvatinib resistance (Fig. 1a). A total of 184 differentially expressed genes were included in the Gene Ontology (GO) enrichment analysis, which suggested that the phosphatidylinositol 3-kinase (PI3K)/protein kinase B (AKT) signaling pathway and mitogen-activated protein kinase (MAPK) signaling pathway may be the key pathways involved in lenvatinib resistance (Fig. 1b). Among the differentially expressed transcripts, ubiquitin proteasome system 14 (USP14) was the most significantly expressed in the lenvatinib-resistant tissues compared with those in the lenvatinib-sensitive tissues (Fig. 1c). Therefore, we next focused on USP14 for further investigation. Quantitative real time polymerase chain reaction (qRT‒PCR) and Western blotting experiments indicated that USP14 expression was significantly elevated in lenvatinib-resistant cell lines (Fig. 1d), as well as in 10 pairs of fresh tumor tissues from resistant patients and sensitive patients (Fig. 1e‒g). Moreover, in HCC patients not treated with lenvatinib, the expression of USP14, as indicated by immunohistochemistry, was higher in tumor samples than in normal samples (Fig. 1h-i and Table S1). Prognostic analysis indicated that elevated USP14 expression was correlated with advanced tumor stage, multiple tumor numbers, and poor prognosis in patients with HCC (cohort 1, n=170) (Fig. 1j-l, and Table S2). Thus, USP14 may be a biomarker indicating resistance to lenvatinib treatment and poor outcomes in HCC patients.

### Loss of USP14 suppresses HCC lenvatinib resistance *in vitro* and *in vivo*

**Figure 2:**
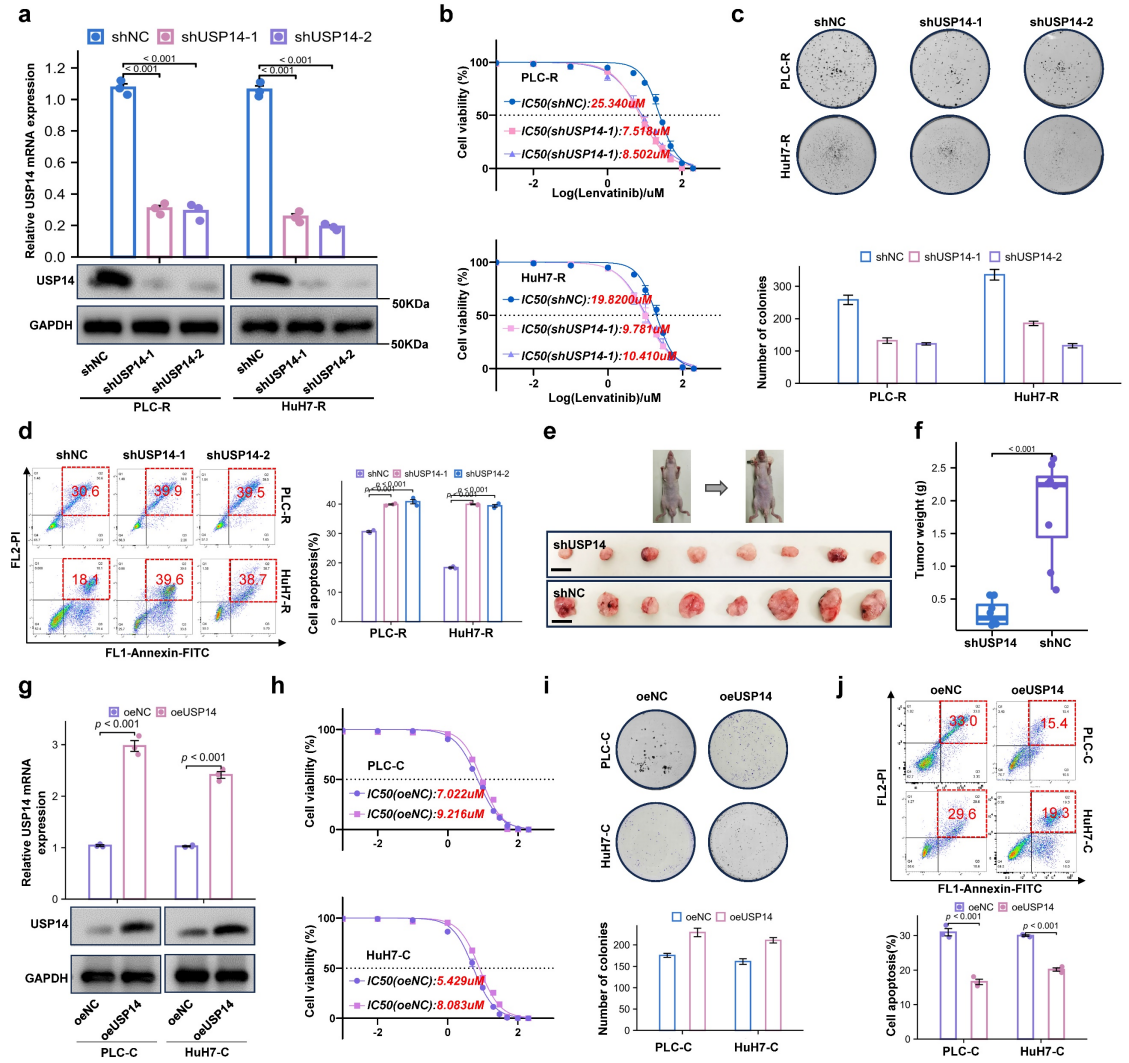
Loss of USP14 suppresses HCC lenvatinib resistance *in vitro* and *in vivo*. **a.** Knockdown of USP14 in the PLC-R and Huh7-R cell lines was confirmed by qRT‒PCR and Western blotting. **b.** CCK-8 assay of USP14-knockdown cell lines and control cell lines treated with lenvatinib at the indicated concentrations for 72 h. **c.** Colony formation assay of lenvatinib-resistant cell lines and shUSP14-treated cells treated with lenvatinib in 6-well plates for 3 weeks (n=3). Representative images (left) and average numbers of colonies (right) are shown. **d.** Analysis of apoptosis in PLC-R and Huh7-R cells treated with lenvatinib by flow cytometry. Representative images (left) and the average number of apoptotic cells (right) are shown. PLC-R cells (5×10^6^ cells per tumor) expressing the control vector or USP14-shRNA were injected into the left and right dorsal flanks of nude mice (n = 8 for each group). Mice were sacrificed 6 weeks after injection. Representative tumor images **e.**, tumor volume and tumor weight **f.** are shown. **g.** Overexpression of USP14 in PLC-C and HuH7-C cell lines was confirmed by qRT-PCR and western blotting. **h.** CCK8 assay of USP14-overexpressed cell lines and control cell lines with lenvatinib treatment at indicated concentrations for 72 h. **i.** Colony formation assay of oeUSP14 group and control group with lenvatinib treatment in 6-well dish for 3 weeks (n=3). Representative images (up) and average number of colonies (down) are shown. **j.** Analysis of apoptosis in oeUSP14 group and control group with lenvatinib treatment by flow cytometry. Representative images (up) and average number of apoptosis (down) are shown. Three independent experiments with three technical repetitions were performed. Student's *t* test was used for statistical analyses. *p* < 0.05 was considered to indicate statistical significance. * *p* < 0.05, ** *p* < 0.01, *** *p* < 0.001.

To further investigate the role of USP14 in the process of lenvatinib resistance, we knocked down USP14 in lenvatinib-resistant cell lines. The knockdown efficiency of shUSP14 was confirmed by qRT‒PCR and Western blotting (Fig. 2a). After knocking down USP14, PLC-R and Huh7-R cells treated with lenvatinib had significantly lower proliferative capacities than those in the control group, as suggested by the IC50 value (Fig. 2b). In addition, there were significantly fewer colonies formed in the shUSP14 group than in the control group (Fig. 2c), while the apoptosis level was significantly increased in the lenvatinib treatment group (Fig. 2d). To investigate the effect of USP14 *in vivo*, the PLC-R-shUSP14 cell line and the control cell line were transplanted into the flanks of nude mice. CDXs were treated with lenvatinib, and the tumor volume was measured weekly beginning on the seventh day after injection (Fig. 2e-f). The growth of the shUSP14 cell-derived tumors in terms of tumor weight (*p*<0.001) was significantly lower compared with that in the control group (Fig. 2f). USP14 was also overexpressed in the PLC-C and Huh7-C cell lines (Fig. 2g). The results suggested that, compared with those in the Control group, the resistance to lenvatinib in the oeUSP14 group was significantly higher, as shown by the IC50 value (Fig. 2h), colonies formed (Fig. 2i) and apoptosis level (Fig. 2j).

### USP14 interacts with and stabilizes CIB1 through deubiquitinase activity

To further elucidate the underlying mechanism through which USP14 regulates lenvatinib sensitivity, USP14-knockdown PLC-R cells were subjected to transcriptome analysis (Fig. S2a). GO and Kyoto Encyclopedia of Genes and Genomes (KEGG) enrichment analyses of the RNA-seq data indicated that USP14 silencing modulated a number of signaling pathways, such as the MAPK, PI3K-AKT and complement and coagulation cascades pathways, in PLR-R cells; among these pathways, MAPK signaling dramatically overlapped with the results of lenvatinib-resistant vs. nonresistant transcriptome sequencing, suggesting that MAPK signaling may be the key pathway mediating USP14-mediated resistance to lenvatinib. (Fig. S2b and Fig. 1b). Gene set enrichment analysis (GSEA) of the RNA-seq data from the TCGA LIHC cohort also indicated that USP14 modulated the MAPK pathway (Fig. S2c). Notably, overexpression of USP14 increased the phosphorylation of MEK1/2 (pMEK1) and phosphorylated ERK1/2 (pERK1/2) in PLC-C and Huh7-C cells (Fig. S2d).

**Figure 3:**
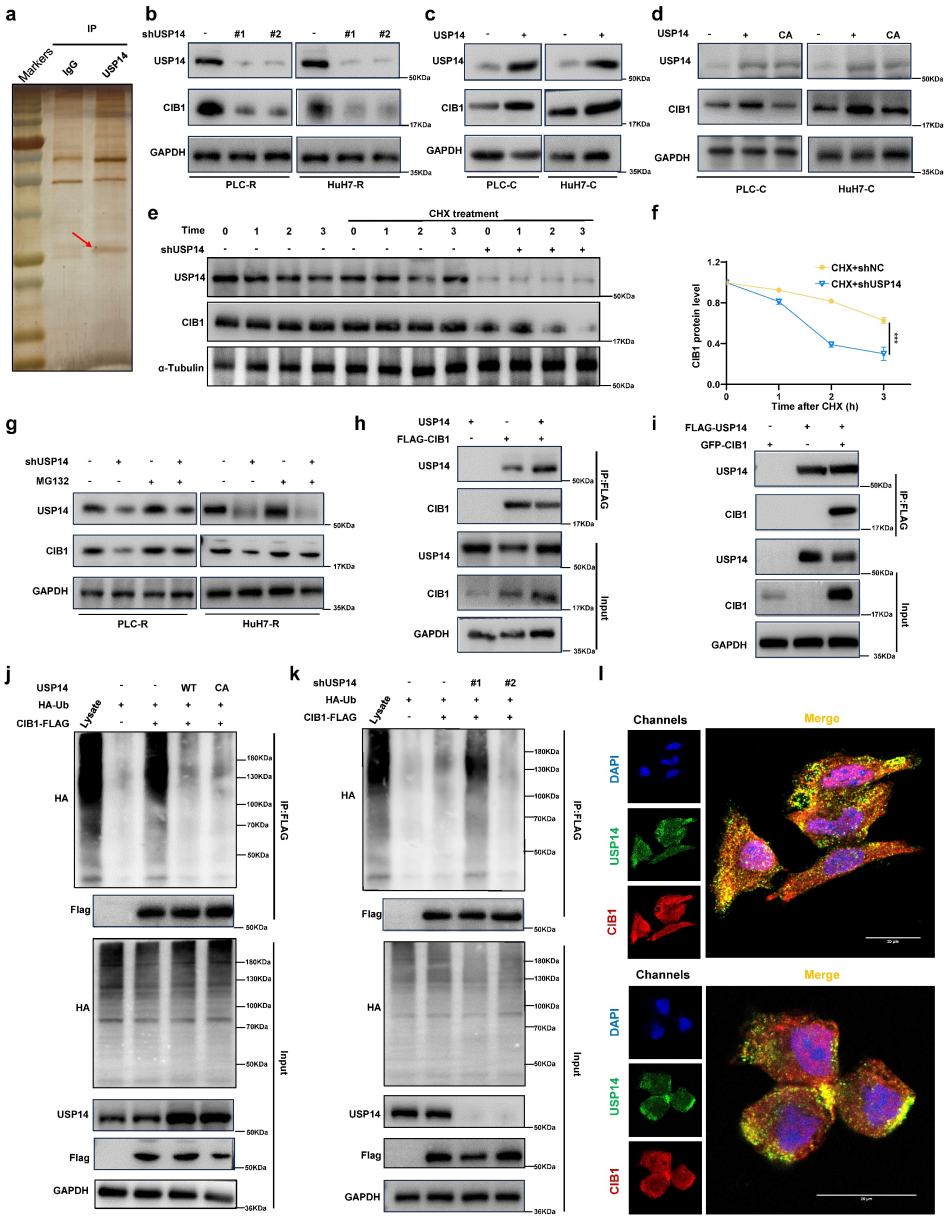
USP14 interacts with and stabilizes CIB1 through deubiquitinase activity. **a.** Silver staining of immunoprecipitated proteins by using USP14/CIB1 or IgG antibodies. **b.** Immunoblotting analysis of CIB1 protein expression in PLC-R cells (left) and Huh7-R cells (right) depleted of USP14 by shRNA. **c.** PLC-C (left) and Huh7-C (right) cells were transfected with USP14. Immunoblotting analysis was performed with the indicated antibodies. **d.** Cells were transfected with control vector, USP14 WT or USP14 CA. CIB1 was upregulated by USP14 WT (Lane 2) but not by USP14 CA (Lane 3). **e-f.** PLC-R cells stably expressing control shRNA or USP14 shRNA were treated with or without cycloheximide (40 µg/mL) and harvested at the indicated times. The protein levels of USP14 and CIB1 were analyzed by immunoblotting and densitometry. **g.** PLC-R cells (left) and Huh7-R cells (right) transfected with the indicated shRNA were left untreated or treated with MG132 (10 μm) for 8 hours, after which the cell lysates were immunoblotted as indicated. **h-i.** Interaction between USP14 and CIB1. PLC-R cells were cotransfected with the indicated constructs. Cellular extracts were immunoprecipitated with FLAG Sepharose and GFP, and immunoprecipitations were performed with antibodies against the indicated proteins. **j.** PLC-C cells transfected with control constructs, USP14(WT) or USP14(C114A) were treated with MG132 (10 mmol/L) for 8 hours before harvesting. Cell lysates were immunoprecipitated with FLAG Sepharose to detect the ubiquitin chains on CIB1. **k.** PLC-R cells stably expressing control, USP14 shRNA#1 or USP14 shRNA#2 were transfected with the indicated constructs and subsequently treated with MG132. Cell lysates were immunoprecipitated and subjected to immunoblotting analysis of ubiquitin. **l.** Immunofluorescence showed the colocalization of USP14 and CIB1 in PLC-R cell lines (upper) and Huh7-R cell lines (lower). Three independent experiments with three technical repetitions were performed. Student's* t* test was used for statistical analyses. *p* < 0.05 was considered to indicate statistical significance. * *p* < 0.05, ** *p* < 0.01, *** *p* < 0.001.

In contrast, USP14 knockdown suppressed the MAPK signaling in PLC-R and Huh7-R cells (Fig. S2d). Moreover, there was a positive correlation between USP14 and p-MEK1/2 (Spearman r¼ 0.562; P < 0.05), or USP14 and p-ERK1/2 (Spearman r ¼ 0.735; n ¼ 40; P < 0.05) after staining for USP14, p-MEK1, and p-ERK1/2 in the tissue of 15 HCC cases (Fig. S2e and f). Subsequently, we investigated how USP14 facilitates the MAPK signaling pathway in HCC. MS analysis of PLC-R cells was performed using anti-USP14 or IgG antibodies (Fig. 3a). We found that CIB1 was the top ranked protein according to the MS data obtained with the anti-USP14 antibody (Table S3; Fig. S3a). We also measured the expression of relevant molecules in our knockdown samples by Western blotting, and the results indicated that the expression of CIB1 was significantly reduced (Fig. 3b). Consistently, the introduction of exogenous USP14 into PLC-C and Huh7-C cells strongly upregulated the protein level of CIB1 (Fig. 3c). To test whether the deubiquitinase activity of USP14 is required for its function in CIB1 regulation, we overexpressed WT or a catalytically inactive mutant (C114A) of USP14 in the PLC-C and Huh7-C cell lines and found that WT USP14, but not the CA mutant, increased the CIB1 protein level (Fig. 3d). Next, cells stably expressing control shRNA or USP14 shRNA were treated with cycloheximide (CHX), a general inhibitor of protein synthesis, and the level of CIB1 decreased significantly after USP14 knockdown under CHX exposure (Fig. 3e-f; Fig. S3b-c). After treating USP14-deficient cells with the proteasome inhibitor MG132, we restored the protein level of CIB1, which was decreased by USP14 depletion (Fig. 3g). We then sought to determine whether USP14 modulates CIB1 by directly interacting with this pair of proteins. We cotransfected HEK293T cells and PLC-R with exogenous CIB1 and USP14, and a coimmunoprecipitation (co-IP) assay confirmed that CIB1 physically interacted with USP14 (Fig. 3h; Fig. S3d). After reciprocal co-IP with anti-Flag antibodies, exogenous USP14 was also found to interact with GFP-CIB1 (Fig. 3i; Fig. S3e). Consistently, endogenous USP14 co-IPed with FLAG-CIB1 as well (Fig. S3f). Furthermore, endogenous interaction between USP14 and CIB1 was confirmed (Fig. S3g). Next, we examined whether USP14 could reverse the polyubiquitination of CIB1. As shown in Fig. 3j, overexpression of USP14, but not of USP14-C114A, significantly reduced the ubiquitination level of CIB1. Consistently, USP14 knockdown markedly increased the level of ubiquitinated CIB1 (Fig. 3k). Immunofluorescence staining of PLC-R and Huh7-R cells also showed the colocalization of USP14 and CIB1 (Fig. 3l). Patients with higher USP14 expression also have higher CIB1 expression levels suggested by immunofluorescence staining of HCC tissues (Fig. S3h-i). These results demonstrated that USP14 directly interacts with and deubiquitinates CIB1.

### K24 is important for K48-linked ubiquitination-mediated CIB1-USP14 interaction

**Figure 4:**
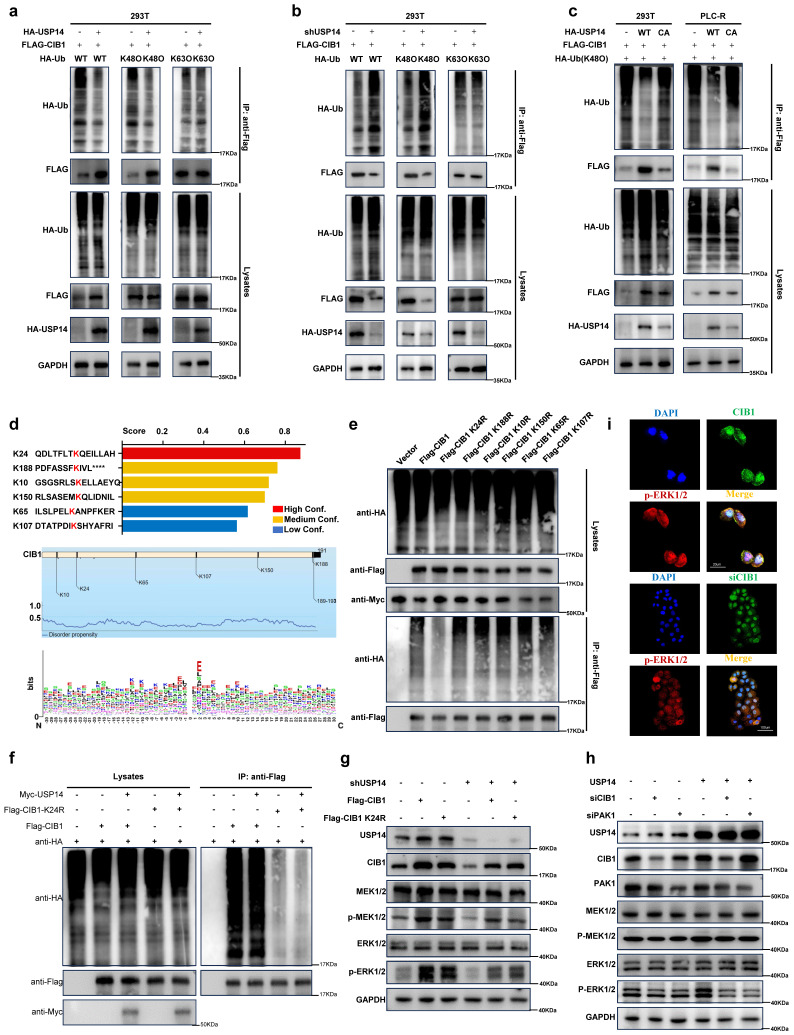
K24 is important for K48-linked ubiquitination-mediated CIB1-USP14 interaction. HEK293T cells transfected with HA-USP14 or the empty vector **(a)** or shNC or shUSP14 **(b)** and cotransfected with FLAG-CIB1 and a vector encoding HA-WT-Ub or its mutants (HA-K48O-Ub or HA-K63O-Ub) were subjected to denaturing-IP and immunoblotted with the indicated antibodies. **c.** PLC-R and Huh7-R cells transfected with the vector plasmid, HA-USP14 or HA-USP44 (C114A) together with FLAG-CIB1 and HA-K48O-Ub were subjected to denaturing-IP and immunoblotted with the indicated antibodies. **d.** The ubiquitination sites of CIB1 were predicted by BDM-PUB and UBPRED. **e.** HEK293T cells were transfected with the indicated plasmid combinations to measure K48-linked ubiquitination of CIB1. **f.** HEK293T cells overexpressing Myc-USP14 or HA-K48 were transfected with the indicated plasmid combinations to measure the ubiquitination of Flag-CIB1/CIB1-K24R. **g.** Western blot showing the effects of CIB1 overexpression on the ERK1/2 pathway in the USP14-knockdown and control groups in PLC-R cells. **h.** Western blot showing the effects of knocking down CIB1 or PAK1 on the ERK1/2 pathway in the USP14 overexpression and control groups in PLC-R cells. **i.** Immunofluorescence showed the effects of CIB1 knockdown or control treatment on the expression of p-ERK1/2 in the PLC-R cell line. Three independent experiments with three technical repetitions were performed. Student's *t* test was used for statistical analyses. *p* < 0.05 was considered to indicate statistical significance. * *p* < 0.05, ** *p* < 0.01, *** *p* < 0.001.

Furthermore, overexpression of USP14 inhibited the K48-linked but not K63-linked ubiquitination of CIB1 in HEK293T cells and PLC-R cells (Fig. 4a; Fig. S4a). Conversely, knockdown of USP14 enhanced the K48-linked but not K63-linked ubiquitination of CIB1 (Fig. 4b; Fig. S4b). In addition, the ability of USP14 (C114A), a deubiquitinase-inactive mutant of USP14, to stabilize and deubiquitinate CIB1 was lost in HEK293T and PLC-R cells (Fig. 4c), which indicates that the ubiquitin hydrolase activity of USP14 is involved in the regulation of CIB1. Based on the intersection predicted by BDM-PUB (http://bdmpub.biocuckoo.org/results.php) (Fig. 4d), six lysine residues (K10, K24, K65, K107, K150 and K188) were selected to construct CIB1 mutants (K to R, respectively). HEK293T cells were transfected with plasmids encoding wild-type (WT) and CIB1 mutants K to R, together with Myc-USP14. The results of a subsequent ubiquitination assay showed that USP14-mediated K48-linked ubiquitination of CIB1 was obviously reduced after mutation of K24 in CIB1 (Fig. 4e). We additionally explored the effect of the CIB1 K24 site mutation on K48-linked ubiquitination. The results of a subsequent ubiquitination assay indicated that the K24 site mutation significantly reduced the K48-linked ubiquitination of CIB1 mediated by USP14 in both HEK293T and PLC-R cells (Fig. 4f; Fig. S4c).

### The CIB1/PAK1 complex is responsible for USP14-induced facilitation of the MAPK pathway

Several signaling pathways, such as the MAPK pathway, are activated during lenvatinib resistance, and our previous results reached the same conclusion (Fig. S2b and Fig. 1b). However, CIB1 itself lacks known enzymatic activity. P21-activated kinase 1 (PAK1) is a validated binding partner of CIB1 that provides important insights into how CIB1 regulates these biological processes. 27 The colocalization and interaction of PAK1 with CIB1 were also demonstrated in two HCC cell lines (Fig. S4d-e). Thus, we wondered whether the CIB1/PAK1 complex functions as a mediator of MAPK pathway facilitation and a protective mechanism for the survival of HCC cells upon exposure to lenvatinib. Indeed, facilitation of the MEK1/2-ERK1/2 cascade is elicited by knockdown of USP14 in two HCC cell lines, as determined by decreases in p-MEK1/2 and p-ERK1/2, while overexpression of CIB1 rescued this process (Fig. 4g; Fig. S4f).

In contrast, treatment with siCIB1 or siPAK1 inhibited the USP14-induced facilitation of the MAPK pathway in HCC cells (Fig. 4h and Fig. S4g). Immunofluorescence also showed that siCIB1 or siPAK1 significantly decreased the expression of p-ERK1/2 at the protein level (Fig. 4i). Thus, these data indicated that the CIB1/PAK1 complex modulates sensitivity to lenvatinib through USP14-induced facilitation of the MAPK pathway.

### USP14 maintains lenvatinib resistance via the CIB1/PAK1-ERK1/2 axis

**Figure 5:**
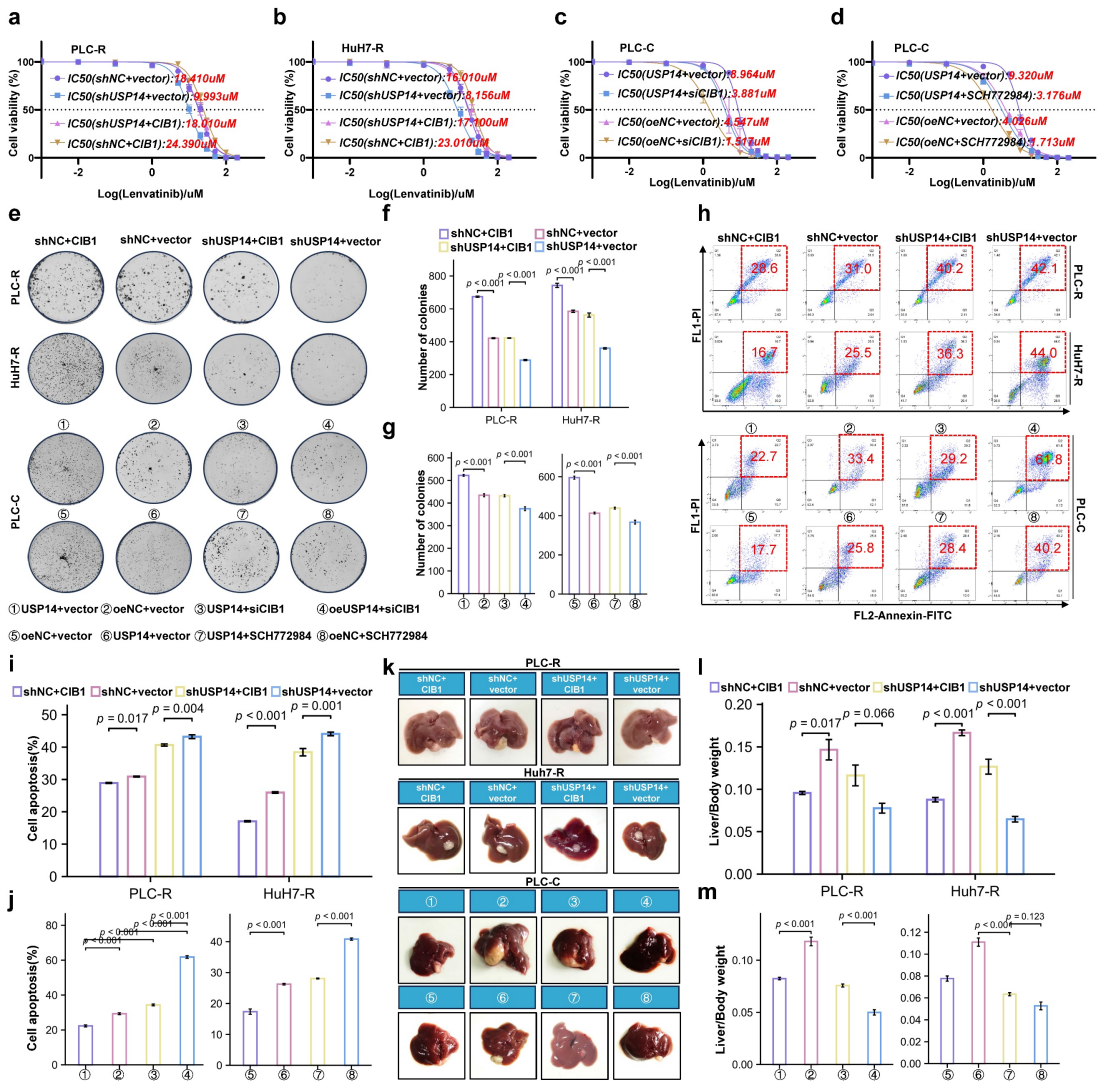
USP14 maintain lenvatinib resistance via CIB1-PAK1/ERK1/2 axis *in vitro* and *in vivo*. **a**-**d**. CCK8 assay of USP14-knockdown but with CIB-overexpression cell lines and control cell lines with lenvatinib treatment at indicated concentrations for 72 h in PLC-R and Huh7-R cell lines. **c**-**d**. CCK8 assay of USP14-overexpression but with CIB1-knockdown cell lines and control cell lines with lenvatinib treatment at indicated concentrations for 72 h in PLC-C and Huh7-C cell lines. **e**-**g**. Representative images and number of colonies of indicated cell lines. **h**-**j**. Representative images and analysis of apoptosis by flow cytometry of indicated cell lines. Representative image **k**. and liver/body weight (%)** l**-**m**. of orthotopic PLC-R or PLC-C tumors treated accordingly. Statistical analyses used Student's t-test. *p* < 0.05 was considered statistically significant. * *p* < 0.05, ** *p* < 0.01, *** *p* < 0.001.

We then performed rescue experiments to validate whether the CIB1-mediated USP14-ERK1/2 pathway plays a critical role in lenvatinib resistance in HCC. In the PLC-R and Huh7-R cell lines, forced expression of CIB1 restored the reduced proliferation of USP14-knockdown cells in the presence of lenvatinib (Fig. 5a-b). Moreover, downregulation of CIB1 impaired USP14-mediated cell proliferation in PLC-C and Huh7-C cells (Fig. 5c-d). Forced expression of CIB1 significantly promoted colony formation by HCC cells after exposure to lenvatinib (Fig. 5e-f). In addition, CIB1 knockdown had a significant effect on the resistance of USP14-overexpressing cells to lenvatinib (Fig. 5g). SCH772984, an inhibitor for ERK1/2, unsurprisingly counteracted the increase in colony formation induced by USP14 (Fig. 5g). Similar results were found for cell apoptosis (Fig. 5h-j), indicating that the CIB1/PAK1-mediated USP14-ERK1/2 pathway promoted lenvatinib resistance *in vitro*. To further examine the effects of USP14 on HCC progression *in vivo*, we constructed xenograft tumor models. Knockdown of USP14 significantly inhibited liver weight and tumor weight in xenograft tumor models, consistent with the results shown in Fig. 2E-F. Overexpression of CIB1 significantly alleviated the USP14 knockdown-induced inhibition of tumor growth and tumor weight (Fig. 5k). After the suppression of CIB1 expression with siCIB1, the liver/tumor weight ratio was significantly lower than that in the corresponding oeUSP14 and oeNC groups (Fig. 5l-m). In addition, SCH772984 enhanced the killing effect of lenvatinib in both the oeNC group and the oeUSP14 group (Fig. 5m). Again, the results indicated that USP14 promotes HCC and maintains lenvatinib resistance via the CIB1/PAK1-ERK1/2 axis.

### Targeting CIB1 *in vivo* retards lenvatinib-resistant HCC

**Figure 6:**
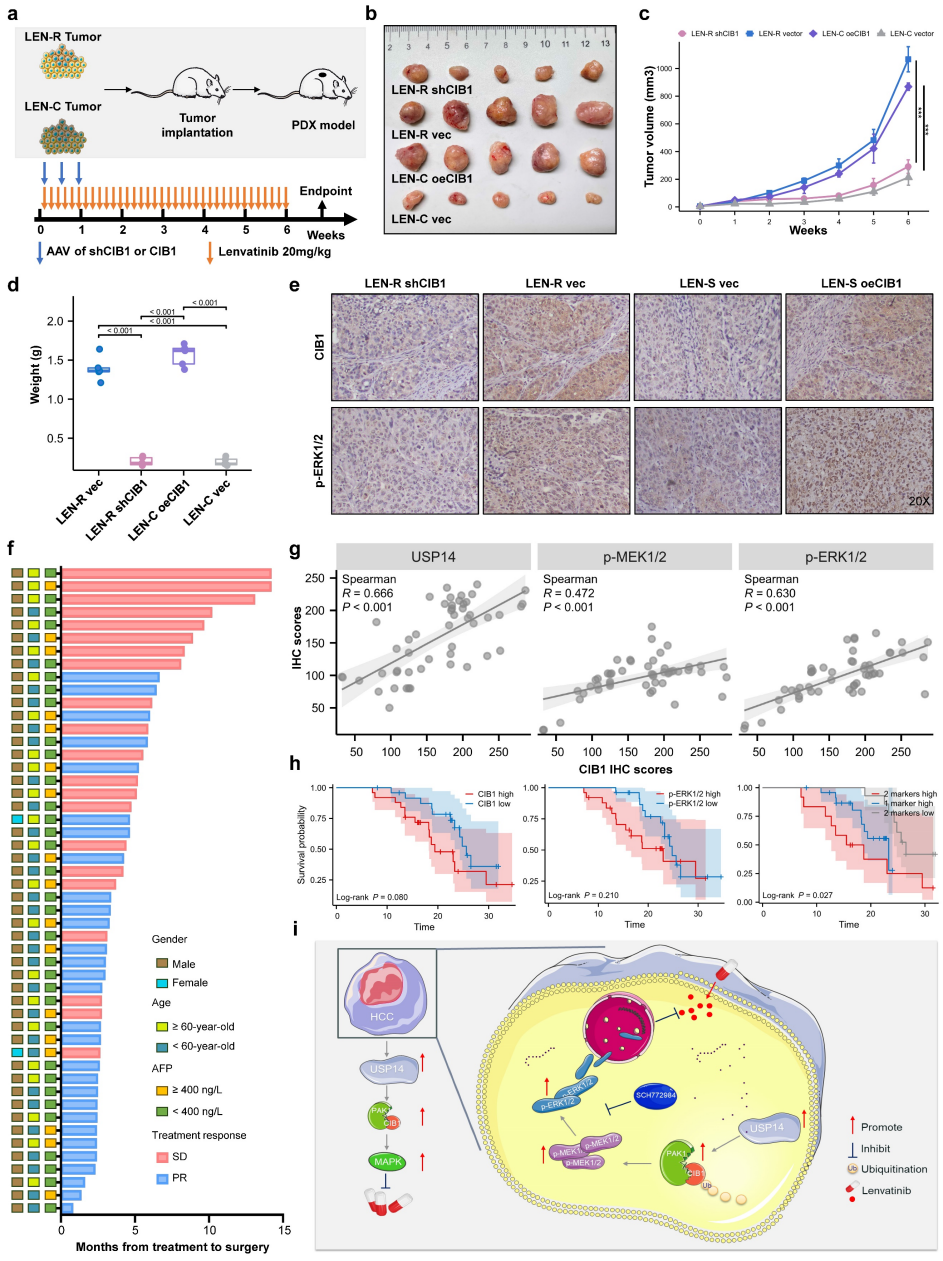
*In vivo* targeting of USP14 retards lenvatinib-resistant HCC. **a.** *In vivo* experimental diagram. **b.** PDXs tumor images after 4 weeks of indicated treatment.** c.** The volume growth curve of the PDX model after 4 different treatments. **d.** PDX tumor weights after 4 weeks of treatment. **e.** IHC analysis of CIB1 and p-ERK1/2 expression in tumor samples from the PDX model. **f.** The cohort of HCC patients who underwent hepatectomy after treatment with lenvatinib; cohort 2; n=50. **g.** Correlation analysis between USP14 and CIB1 expression, p-ERK1/2 and CIB1 expression, and p-MEK1/2 and CIB1 expression in HCC tissues from cohort 2. **h.** Survival was determined and compared between patients with high and low CIB1 expression and p-ERK1/2 expression in the HCC tissues of cohort 2; log-rank test was used.** i**. Proposed working model of USP14. Three independent experiments with three technical repetitions were performed. Student's *t* test was used for statistical analyses. *p* < 0.05 was considered to indicate statistical significance. * *p* < 0.05, ** *p* < 0.01, *** *p* < 0.001.

To further verify our findings and clinical significance *in vivo*, we transplanted tumor specimens from lenvatinib-resistant patients into immunodeficient mice. After sufficiently long passages of treatment with lenvatinib, patients who were evaluated as PD or SD were considered lenvatinib-resistant patients. The patient-derived xenograft (PDX) mouse model was treated with lenvatinib after local injection of adeno-associated virus 9 (AAV)-mediated shCIB1/oeCIB1 or its negative control (Fig. 6a). Tumor volume was measured weekly beginning on the seventh day after injection. Local injection of shCIB1 AAV into the subcutaneous implantation site of lenvatinib-resistant PDX mice significantly restored the sensitivity of resistant tumor cells to lenvatinib, as suggested by the tumor volume and tumor weight (Fig. 6b-d). In contrast, local injection of AAV from OE-CIB1 promoted lenvatinib resistance in the PDX-S models (Fig. 6b-d). Immunohistochemical (IHC) staining was performed on PDX tumor samples to assess the influence of expression of CIB1 on the MAPK pathway (Fig. 6e).

In a novel cohort of HCC patients receiving preoperative therapy, including lenvatinib (cohort 2, n = 50) (Fig. 6f), the IHC results indicated that USP14 expression was positively correlated with CIB1 expression in HCC tissues (Fig. 6g). A summary of the clinical characteristics of all patients is provided in Table S4. Correlation analysis also indicated that high CIB1 expression was positively related to high expression of p-MEK1/2 and p-ERK1/2 and vice versa (Fig. 6g). We next explored the correlation of overall survival in cohort 3 by evaluating the associations between CIB1 expression and p-ERK1/2 levels. In cohort 3, patients with high CIB1 or p-ERK1/2 expression had worse prognoses than those with low CIB1 or p-ERK1/2 expression (Fig. 6h). The shortest overall survival times were observed for patients with both increased CIB1 and p-ERK1/2 expression (Fig. 6h). Overall, these data indicate that high expression levels of MAPK markers may identify HCC patients with poor prognosis and that targeting CIB1 may be a critical pharmaceutical intervention for lenvatinib monotherapy or combination therapy.

## Discussion

Current treatment options for advanced HCC include targeted therapy and immunotherapy. The median survival time for patients receiving lenvatinib, a first-line targeted therapy worldwide, was only 10-15 months. The objective response rates of these patients were 42.1% in Japan,^28^ 22.2% in China,^29^ and 18.9% in Korea.^30^ Our previous study showed that more than 70% of patients receiving lenvatinib combined therapy were nonresponders according to Response Evaluation Criteria in Solid Tumors (RECIST) v1.1.^31^ Treatment resistance is one of the main factors leading to the high mortality rate of HCC patients, but the underlying mechanism has not been fully analyzed. It has been reported that cancer-associated fibroblast-derived secreted phosphoprotein 1 enhances TKI resistance in HCC through bypassing the activation of carcinogenic signaling and promoting the EMT, activating MAPK and PI3K/AKT mammalian target of rapamycin (mTOR).^32^ Leung *et al.* demonstrated CDK6 as a druggable target in lenvatinib-resistant HCC. They identified a noncanonical pathway of CDK6, leading to activation of Wnt/β-catenin signaling.^33^ The current study rigorously confirmed that USP14 is the key molecule involved in promoting lenvatinib resistance. Mechanistically, the deubiquitinating function of USP14 stabilizes CIB1 at K24, which in turn interacts with PAK1 and promotes the MAPK pathway and lenvatinib resistance (Fig. 6i). Clinically, high CIB1 expression was significantly correlated with poor overall survival in patients with HCC complicated with lenvatinib resistance. These data strongly suggested that CIB1 might serve as an oncogene in HCC tumorigenesis and in lenvatinib-resistant HCC cells.

To investigate the various aspects of the mechanism of lenvatinib resistance, we established a lenvatinib resistance model *in vivo* by continuous intragastric administration as the main functional experimental vector. Compared to the methods of adding lenvatinib directly to cell culture reported by others,^26^, ^34^ we believe that the *in vivo* model is more rigorous because the development of resistance involves complex kinase and signaling pathway regulation *in vivo*. We also collected a large number of HCC specimens, including treatment-naive and lenvatinib-resistant specimens, to analyze the characteristics of lenvatinib resistance in multiple dimensions of HCC. Encouragingly, we identified a novel lenvatinib resistance-related molecule, USP14. Our results indicate that USP14 is significantly associated with poor prognosis in HCC patients and resistance to lenvatinib both *in vitro* and *in vivo*. Human USP14 contains a total of 494 amino acids and can be divided into two regions based on its function. One is the N-terminal ubiquitin-like domain (UBL, approximately 9 kDa), and the other is the C-terminal catalytic domain (USP, approximately 45 kDa). The UBL domain can enhance the activity of various enzymes in the proteasome, while the USP domain is closely related to deubiquitinase activity.^35^ We discovered that the CIB1 molecule interacts with USP14 using mass spectrometry, but we do not know whether USP14 modifies CIB1 through its deubiquitinating activity. Follow-up studies further confirmed that in HCC cells, USP14 indeed maintains the stability of CIB1 through K48-linked deubiquitination in a catalytic activity-dependent manner.

CIB1 is a widely expressed calcium-binding protein that interacts with a variety of signaling proteins. CIB1 plays a regulatory role in many cellular processes, such as cell differentiation, cell division, cell proliferation, cell migration, thrombosis, angiogenesis, cardiac hypertrophy, and apoptosis.^36^, ^37^ Our results highlight the important role of CIB1 in influencing the resistance of HCC to lenvatinib. The occurrence of drug resistance involves the activation of multiple signaling pathways. Therefore, we speculate that CIB1 affects lenvatinib resistance in HCC by affecting certain pathways.^38^ Previous studies have shown that lenvatinib gradually fails to fully inhibit MAPK signaling during administration, which limits the drug response.^25^ Thus, MAPK pathway activation demonstrates the adverse consequences of lenvatinib therapy. Our comparison of the resistant group and the nonresistant group and USP14 transcriptome both strongly suggested that MAPK is a key signaling pathway involved in the development of lenvatinib resistance, which is mediated by CIB1/PAK1 complex, as inhibiting PAK1 also blocks the effect of CIB1 on ERK1/2 signaling. Our observation that silencing the MAPK pathway also inhibits HCC lenvatinib resistance further confirmed that CIB1 promotes HCC resistance through the PAK1-ERK1/2 axis.

Interfering with CIB1 levels during host tumor progression in the presence of lenvatinib in both *in situ* liver tumor and PDX models and high CIB1 expression predict significantly poor postoperative survival in a neoadjuvant cohort. The combination of CIB1 deletion with docetaxel or TRAIL selectively and frequently enhances the death of primary and docetaxel-resistant TNBC cells while preserving normal cells. 39 Thus, combination therapy targeting CIB1 may prove to be a safe and durable strategy for treating triple-negative breast cancer and potentially other cancers.^39^ Our research highlights the value of CIB1 blockade as an effective candidate to overcome HCC resistance to lenvatinib. Although small-molecule compounds have been suggested to be potential therapeutics for HCC, several concerns, such as the lack of specific effects on tumor enzymes vs. normal cell enzymes, have yet to be resolved.

## Conclusions

In summary, USP14-mediated modulation of the MAPK pathway represents a potential mechanism by which HCC cells develop resistance to lenvatinib. By stabilizing CIB1 through deubiquitination, which facilitates the MAPK signaling cascade, USP14 enables sustained signaling despite the presence of lenvatinib, ultimately promoting cancer cell survival and proliferation. Understanding the intricate interplay between USP14, ubiquitination, and the MAPK pathway is crucial for the development of novel therapeutic strategies to overcome lenvatinib resistance in HCC patients.

## Supplementary Material

Supplementary figures and tables.

## Abbreviations

AAV: adeno-associated virus
CR: complete response
CDX: cell-derived xenograft
CIB1: calcium- and integrin-binding protein 1
GO: Gene Ontology
HCC: hepatocellular carcinoma
LEN-C: lenvatinib control
LEN-R: lenvatinib resistant
PAK1: P21-activated kinase 1
PD: progressive disease
PDX: patient-derived xenograft
PR: partial response
RECIST: Response Evaluation Criteria in Solid Tumors
SD: stable disease
USP14: ubiquitin specific protease 14
