# Supplementary material for: Deubiquitination of CIB1 by USP14 promotes lenvatinib resistance via the PAK1-ERK1/2 axis in hepatocellular carcinoma
Source: Int J Biol Sci. 2024 Jun 3;20(9):3269–84. doi: 10.7150/ijbs.96031 (PMC11234209; doi:10.7150/ijbs.96031)
Supplement: Supplementary file 1 — Supplementary figures and tables. [file ijbsv20p3269s1.pdf]

Supplementary Data

Deubiquitination of CIB1 by USP14 promotes lenvatinib resistance via the PAK1-ERK1/2 axis in hepatocellular carcinoma

Ming-Hao Xu, Yi-Min Zheng, Bu-Gang Liang, Wen-Xin Xu, Jun Cao, Pei Wang, Zi-Ying Dong, Chen-Hao Zhou, Hui-Chuan Sun, Ning Ren, Ai-Wu Ke, and Ying-Hao Shen

The Supplementary Data consist of:

Supplementary Figures 1-4

Supplementary Tables 1-6

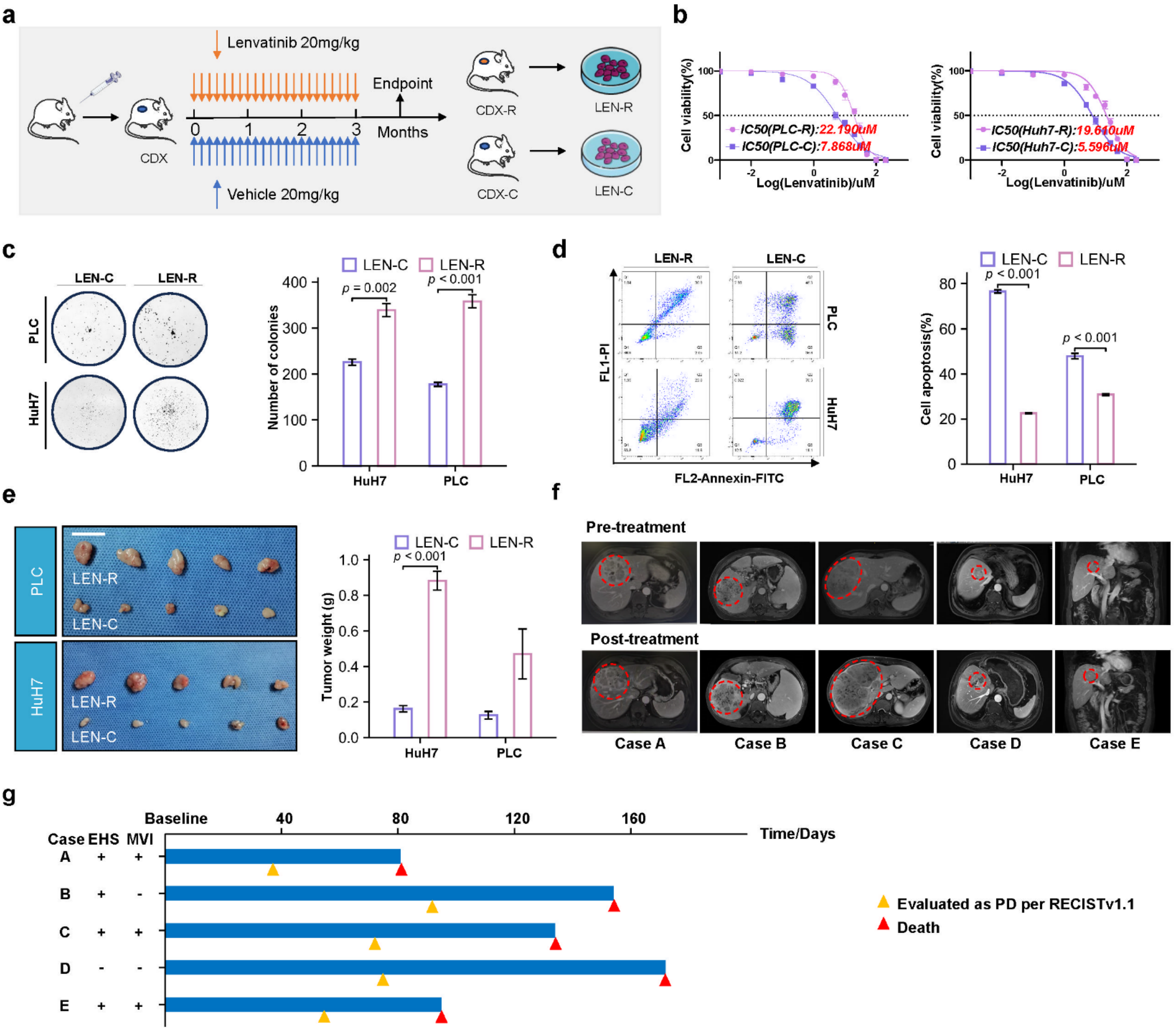

Fig. S1. Establishment and verification of lenvatinib-resistant models. **a.** The graphical representation of lenvatinib-resistant models. **b.** CCK8 assay of lenvatinib-resistant cell lines and control cell lines with lenvatinib treatment at indicated concentrations for 72 h. **c.** Colony formation assay of lenvatinib-resistant cell lines and control cell lines with lenvatinib treatment in 6-well dish for 2 weeks (n=3). Representative images (left) and average number of colonies (right) are shown. **d.** Analysis of apoptosis of lenvatinib-resistant cell lines and control cell lines with lenvatinib treatment by flow cytometry. **e.** Representative pictures and tumor weight of LEN-C and LEN-R under lenvatinib or vehicle treatment (20 mg/kg/day) for 30 days. **f.** Pre- and post-lenvatinib-treatment images of 5 HCC cases. Pre-treatment biopsy specimens were abstained for transcriptome sequencing. **g.** Swimmer plots of the 5 lenvatinib-resistant cases.Three

independent experiments with three technical repetitions were performed. Statistical analyses used Student's *t*-test. CDX, cell-derived xenograft; LEN-R, lenvatinib resistant; LEN-C, lenvatinib control; EHS, extrahepatic spread; MVI, macrovascular invasion; PD, progressive disease; RECIST, Response Evaluation Criteria in Solid Tumors. $p < 0.05$ was considered statistically significant. * $p < 0.05$, ** $p < 0.01$, *** $p < 0.001$.

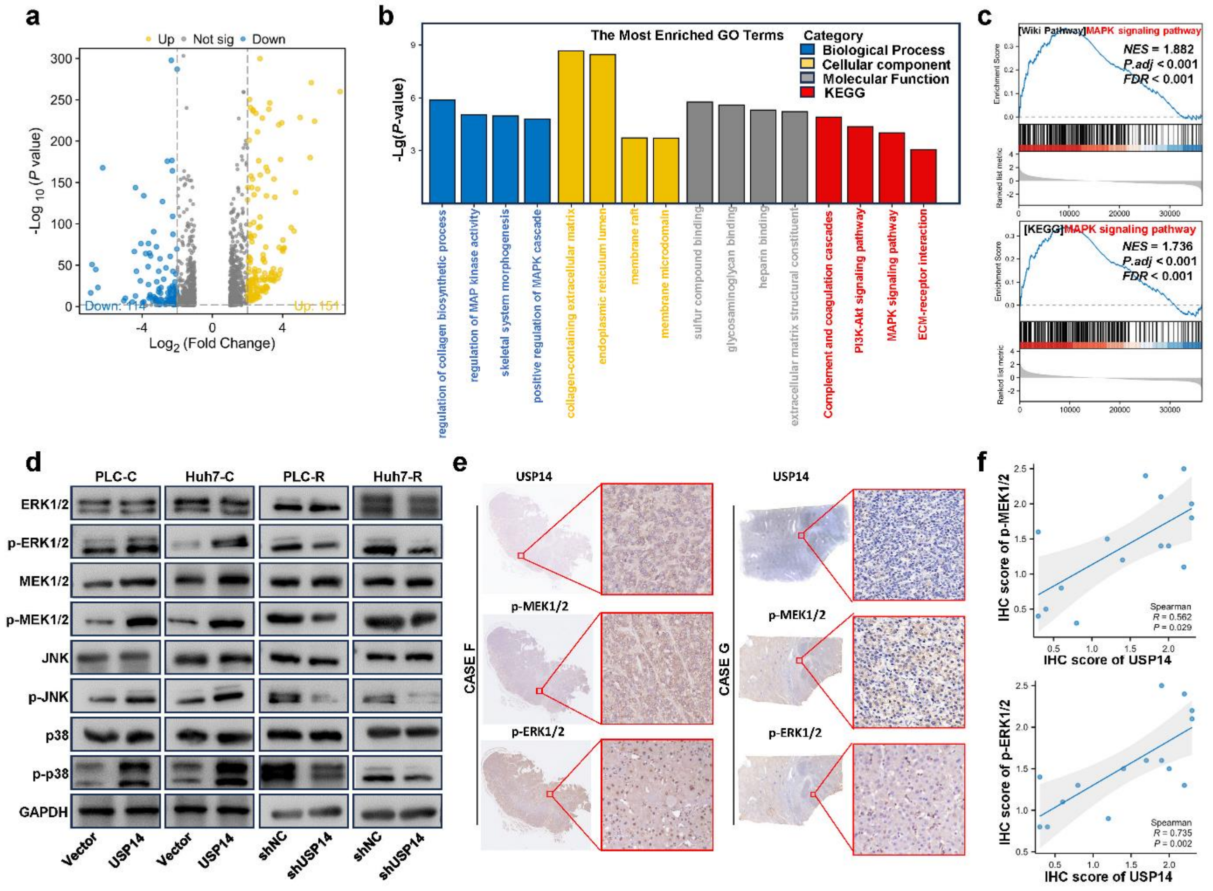

Fig. S2. USP14 promotes the activation of MAPK pathway. **a.** The transcriptome analysis of PLC-R cells of Control group and shUSP14 group. **b.** Gene ontology analysis of differential expressed genes. **c.** GSEA analysis of the TCGA-LIHC dataset after dividing the patient specimens into USP14 high expression and low expression group. **d.** Immunoblot assays of phosphorylated or non-phosphorylated ERK1/2, MEK1/2, JNK, and p38 proteins in the indicated HCC cells. **e-f.** The tissue of 15 HCCs was stained with USP14, p-ERK1/2, or p-MEK1/2 by using the IHC assay. The typical images of IHC are shown in **e.** The expression and correlation of p- MEK1/2 and USP14, or p-ERK1/2 and USP14 are shown in **f.** Three independent experiments with three technical repetitions were performed. *p* values as indicated.

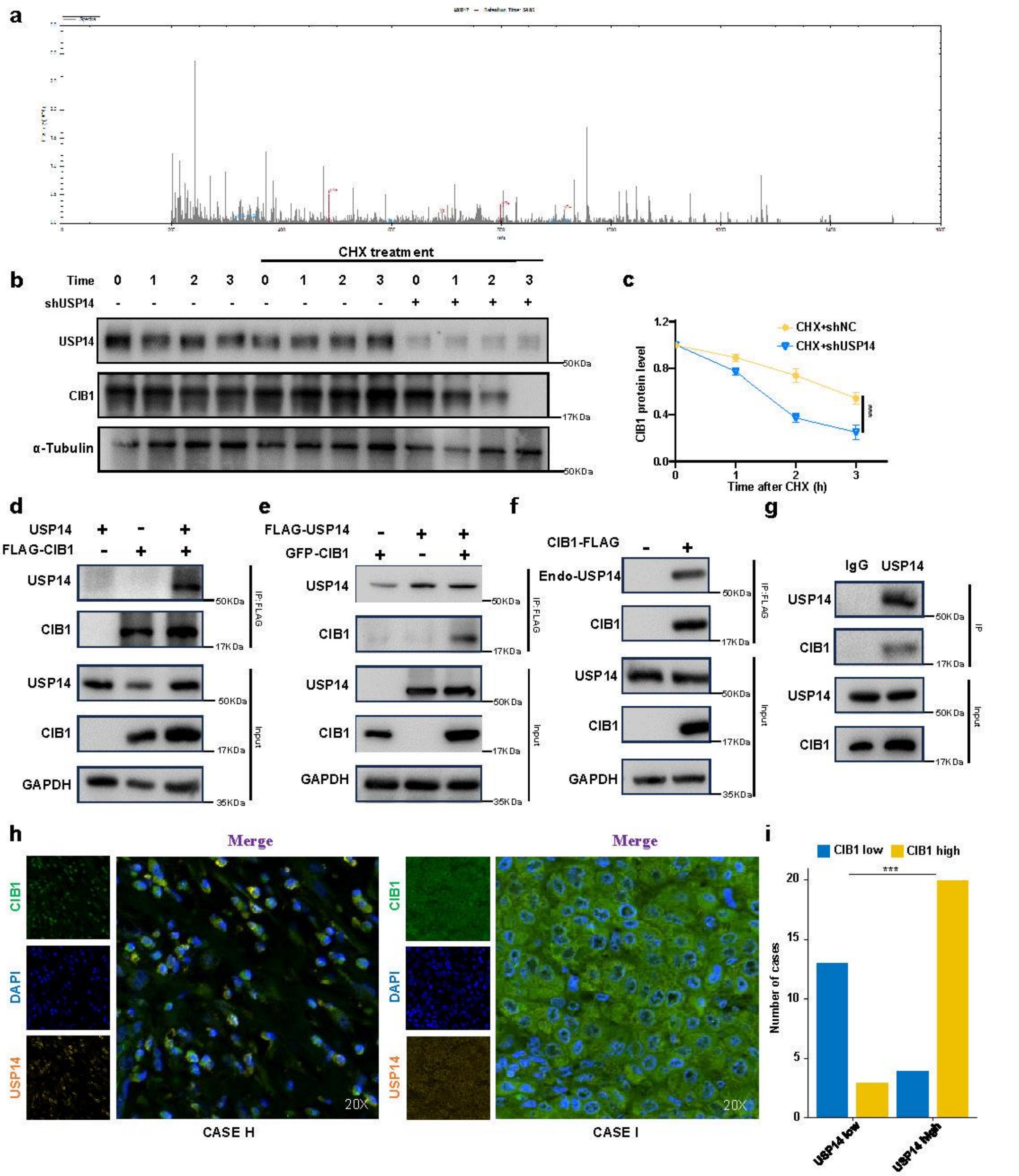

Fig. S3. USP14 interacts with and stabilizes CIB1 through the deubiquitinase activity. **a.** The peptide information of CIB1 in the MS of USP14 in PLC-R cells. **b-c.** Huh7-R cells stably expressing control shRNA or USP10 shRNA were treated with or without cycloheximide (40 ug/mL) and harvested at the indicated times. Protein levels of USP10 and CIB1 were analyzed by immunoblotting and by densitometry. **d-e.** Interaction between exogenous USP14 and CIB1. Huh7-R cells were co-transfected with indicated constructs. Cellular extracts were immunoprecipitated with FLAG Sepharose and immunoprecipitations were performed with antibodies against the indicated proteins. **f.** PLC-R cells were transfected with FLAG-tagged CIB1. Extracts were immunoprecipitated with FLAG Sepharose and examined by immunoblotting.

Endogenous USP14 was detected to interact with CIB1. **g.** Endogenous CIB1 was immunoprecipitated with USP14 antibody and examined by immunoblotting in PLC-R cell lines. **h.** Immunofluorescence showed the co-localization of USP14 and CIB1 in HCC tissues. **i.** Statistical result for the expression of USP14 and CIB1. Three independent experiments with three technical repetitions were performed. *** $p < 0.001$, Fischer's exact test (two sided).

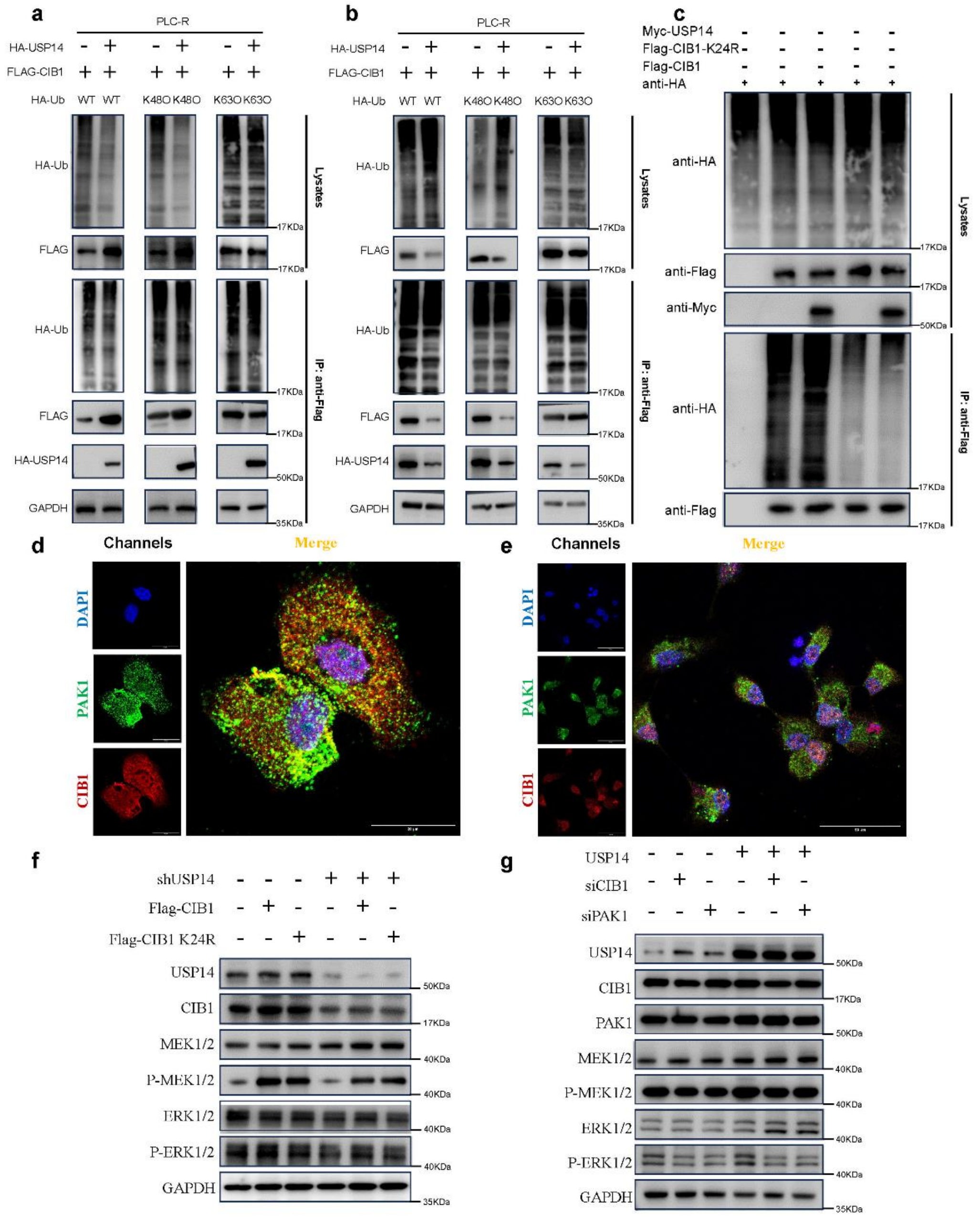

Fig. S4. K24 is important for K48-linked ubiquitination mediated CIB1-USP14 interaction. PLC-R cells transfected with HA-USP14 or the empty vector **a**. and shNC or shUSP14 **b.** co-transfected with FLAG-CIB1 and a vector encoding HA-WT-Ub or its mutants (HA-K48O-Ub or HA-K63O-Ub) were subjected to denature-IP and immunoblotted with the indicated antibodies. **c.** PLC-R cells overexpressing Myc-

USP14 and HA-K48 were transfected with the indicated plasmid combinations to measure the ubiquitination of Flag-CIB1/CIB1-K24R. **d-e.** Immunofluorescence showed the co-localization of PAK1 and CIB1 in PLC-R and Huh7-R cell lines. **f.** Western blot showing the effects of overexpression of CIB1 on ERK1/2 pathway in the USP14-knockdown and control groups in Huh7-R cell lines. **g.** Western blot showing the effects of knocking down CIB1 or PAK1 on ERK1/2 pathway in the USP14 overexpression and control groups in Huh7-R cell lines. Three independent experiments with three technical repetitions were performed.

Supplementary Tables

Table S1. Correlation between USP14 expression and clinicopathological features in HCC patients.

Characteristics	USP14 high (n=83)	USP14 low (n=87)	P value	
Age, years, n (%)			0.297	Chisq test
<60	56 (32.9%)	65 (38.2%)		
≥60	27 (15.9%)	22 (12.9%)		
Gender, n (%)			0.534	Chisq test
Male	75 (44.1%)	76 (44.7%)		
Female	8 (4.7%)	11 (6.5%)		
AFP, ng/mL, n (%)			0.093	Chisq test
>400	33 (19.4%)	24 (14.1%)		
≤400	50 (29.4%)	63 (37.1%)		
HBsAg, n (%)			0.459	Chisq test
Positive	66 (38.8%)	73 (42.9%)		
Negative	17 (10%)	14 (8.2%)		
HCV, n (%)			0.261	Yates' correction
Negative	83 (48.8%)	84 (49.4%)		
Positive	0 (0%)	3 (1.8%)		
TB, umol/L, n (%)			0.532	Chisq test
>21	9 (5.3%)	7 (4.1%)		
≤21	74 (43.5%)	80 (47.1%)		
ALB, g/L, n (%)			0.559	Chisq test
>35	74 (43.5%)	75 (44.1%)		

Characteristics	USP14 high (n=83)	USP14 low (n=87)	P value	
≤35	9 (5.3%)	12 (7.1%)		
ALT, U/L, n (%)			0.283	Chisq test
>40	38 (22.4%)	47 (27.6%)		
≤40	45 (26.5%)	40 (23.5%)		
PT, s, n (%)			0.516	Chisq test
≤14	75 (44.1%)	81 (47.6%)		
>14	8 (4.7%)	6 (3.5%)		
Cirrhosis, n (%)			0.097	Chisq test
No	39 (22.9%)	30 (17.6%)		
Yes	44 (25.9%)	57 (33.5%)		
MVI, n (%)			0.480	Chisq test
No	59 (34.7%)	66 (38.8%)		
Yes	24 (14.1%)	21 (12.4%)		
Differentiation grade, n (%)			0.303	Chisq test
I-II	51 (30%)	60 (35.3%)		
III-IV	32 (18.8%)	27 (15.9%)		
Tumor number, n (%)			0.004	Chisq test
Single	41 (24.1%)	62 (36.5%)		
Multiple	42 (24.7%)	25 (14.7%)		
Tumor size, cm, n (%)			0.110	Chisq test
≤5	58 (34.1%)	70 (41.2%)		
>5	25 (14.7%)	17 (10%)		
BCLC stage, n (%)			0.001	Chisq test
0-A	58 (34.1%)	78 (45.9%)		

Characteristics	USP14 high (n=83)	USP14 low (n=87)	P value
B-C	25 (14.7%)	9 (5.3%)	

Abbreviations: AFP, α-fetoprotein; ALB, albumin; BCLC, Barcelona Clinic Liver Cancer; HBsAg, hepatitis B surface antigen; HCV, Hepatitis C; MVI, microvascular invasion; PT, prothrombin time; TB, total bilirubin; USP14, ubiquitin proteasome system.

Table S2. Univariate and multivariate COX analysis of OS and RFS in 1 cohort (n = 170)

Characteristics		n	Univariate analysis		Multivariate analysis	
			Hazard ratio (95% CI)	P value	Hazard ratio (95% CI)	P value
USP14 high expression	(OS)	83	1.820 (1.095 - 3.026)	**0.021**	1.489 (0.882 - 2.511)	0.136
	(RFS)	83	1.875 (1.214 - 2.897)	**0.005**	1.548 (0.987 - 2.428)	0.057
Single tumor	(OS)	103	0.284 (0.171 - 0.472)	< **0.001**	0.404 (0.212 - 0.768)	**0.006**
	(RFS)	103	0.303 (0.193 - 0.474)	< **0.001**	0.333 (0.198 - 0.558)	< **0.001**
Tumor size >5cm	(OS)	42	1.839 (1.077 - 3.139)	**0.026**	1.863 (1.037 - 3.347)	**0.037**
	(RFS)	42	1.134 (0.691 - 1.859)	0.619		
BCLC stage B-C	(OS)	34	5.209 (3.051 - 8.893)	< **0.001**	2.098 (1.034 - 4.260)	**0.040**
	(RFS)	34	2.483 (1.475 - 4.180)	< **0.001**	1.049 (0.569 - 1.933)	0.879
Differentiation grade I-II	(OS)	111	0.444 (0.270 - 0.733)	**0.001**	0.541 (0.324 - 0.906)	**0.019**
	(RFS)	111	0.726 (0.463 - 1.138)	0.163		
AP>400 ng/mL	(OS)	57	2.023 (1.222 - 3.350)	**0.006**	1.613 (0.950 - 2.738)	0.077
	(RFS)	57	2.107 (1.364 - 3.255)	< **0.001**	1.877 (1.198 - 2.939)	**0.006**

Abbreviations: AFP, α-fetoprotein; BCLC, Barcelona Clinic Liver Cancer; USP14, ubiquitin proteasome system.

Table S3. Top 10 protein scores from 225 unique proteins that interact with USP14 by LC-MS/MS analysis.

Protein names	Gene names	Scores	Diff Sig.
Calcium And Integrin-Binding Protein 1	CIB1	28.510	++
Tubulin Beta 3 Class III	TUBB3	24.771	++
Poly (ADP-Ribose) Polymerase 1	PARP1	20.260	++
Filamin A	FLNA	18.834	++
Protein Kinase, DNA-Activated, Catalytic Subunit	PRKDC	18.029	++
Chromobox 3	CBX3	17.659	++
Synuclein Alpha	SNCA	16.431	++
Proteasome 26S Subunit, Non-ATPase 3	PSMD3	15.881	++
Drebrin 1	DBN1	15.309	++
Solute Carrier Family 1 Member 5	SLC1A5	13.980	++

Table S4. Characteristics of the 50 HCC Patients HCC patients receiving Preoperative-Lenvatinib Therapy, Before Hepatectomy.

Characteristic	Preoperative-lenvatinib-receiving HCC Patients (n)
BCLC stage (A/B/C) (Before lenvatinib-treatment)	5/28/17
China liver cancer stage (Ib/IIa/IIb/IIIa/IIIb) (Before lenvatinib-treatment)	5/13/15/16/1
ECOG performance status (0/1/2)	31/19/0
Child-Pugh class (A/B)	50/0
Tumor response, according to RECIST v1.1 (CR/PR/SD/PD)	0/29/21/0
Tumor response, according to mRECIST (CR/PR/SD/PD)	5/31/14/0
Vascular Tumor Thrombus (Yes/No)	16/34
Pathologic Complete Response (Yes/No)	4/46

Abbreviations: BCLC, Barcelona Clinic Liver Cancer; CR, complete response; ECOG, Eastern Cooperative Oncology Group; PD, progressive disease; PR, partial response; SD, stable disease.

Table S5. The shRNA sequence, siRNA sequence and primers in the article.

Gene	Sequence
USP14-shRNA#1	5′-CCCAAGATTCAGCAGTCAGAT-3′
USP14-shRNA#2	5′-GCAGCCAAATACAAGTGACAA-3′
siCIB1	5′-AAGCAGGAGATCCTCCTAGCC-3′
siPAK1	5′-AAGGTTGACATCTGGTCCCTG-3′
siNC	5′-UUCUCCGAACGUGUCACGUTT-3′
Primer-USP14	5′-ATGCCACTCTACTCTGTTACAGT-3′
Primer-GAPDH	5′-GTCTCCTCTGACTTCAACAGCG-3′

Table S6. Primary antibodies used in the study.

Name	Application	Supplier	Cat no.
Rabbit anti-USP14	WB, IHC, IF	Abcam	ab235960
Rabbit anti-CIB1	WB, IF	Abcam	ab220606
Rabbit anti-CIB1	IHC	Abcam	ab198845
Rabbit anti-MEK1/2	WB, IHC	CST	4694
Rabbit anti-p-MEK1/2	WB	CST	9154
Rabbit anti-ERK1/2	WB	CST	9102
Rabbit anti-p-ERK1/2	WB	CST	9101
Rabbit anti-p-ERK1/2	IHC, IF	Proteintech	28733-1-AP
Rabbit anti-PAK1	WB, IF	CST	2602
Rabbit anti-human p38 MAPK	WB	CST	8690
Rabbit anti-human p-p38 MAPK	WB	CST	4511
Rabbit anti-human SAPK/JNK Antibody	WB	CST	9252
Rabbit anti-human p-SAPK/JNK	WB	CST	4668
K48-linkage Specific Polyubiquitin Antibody	WB	CST	4289
Rabbit anti-HA	WB	Sigma	H6908
Rabbit anti-Flag	WB	Sigma	F7425
Mouse anti-Myc-Tag	WB	CST	2276
Rabbit anti-GAPDH	WB	Abcam	ab9458
Rabbit anti-α-Tubulin	WB	CST	2125
